# Personality influences the neural responses to viewing facial expressions of emotion

**DOI:** 10.1098/rstb.2010.0362

**Published:** 2011-06-12

**Authors:** Andrew J. Calder, Michael Ewbank, Luca Passamonti

**Affiliations:** 1 MRC Cognition and Brain Sciences Unit, 15 Chaucer Road, Cambridge CB2 7EF, UK; 2 Consiglio Nazionale delle Ricerche, Unità Ricerca Neuroimmagini, Catanzaro, Italy

**Keywords:** facial expressions, personality, fMRI, amygdala, anxiety, aggression

## Abstract

Cognitive research has long been aware of the relationship between individual differences in personality and performance on behavioural tasks. However, within the field of cognitive neuroscience, the way in which such differences manifest at a neural level has received relatively little attention. We review recent research addressing the relationship between personality traits and the neural response to viewing facial signals of emotion. In one section, we discuss work demonstrating the relationship between anxiety and the amygdala response to facial signals of threat. A second section considers research showing that individual differences in reward drive (behavioural activation system), a trait linked to aggression, influence the neural responsivity and connectivity between brain regions implicated in aggression when viewing facial signals of anger. Finally, we address recent criticisms of the correlational approach to fMRI analyses and conclude that when used appropriately, analyses examining the relationship between personality and brain activity provide a useful tool for understanding the neural basis of facial expression processing and emotion processing in general.

## 1. Introduction

Over the past 15 years, there has been an explosion of interest in the neuropsychological basis of human emotion. A large body of this research has addressed the neural mechanisms underlying the recognition of human signals of emotion, of which the face is the primary source. Research from patients with brain lesions and neuroimaging studies have vastly improved our understanding of the relevant functional and neural mechanisms. The main findings are summarized in Haxby *et al*.'s [1] model of face perception, which is divided into ‘core’ and ‘extended’ systems. The core face system supports the perception of different facial characteristics and is located in occipitotemporal cortex. It comprises separate regions or routes contributing to the perception of ‘changeable’ facial properties, such as facial expressions and eye gaze, and ‘invariant’ facial properties, such as facial identity; although see Calder & Young [2] and Calder [3] for a different perspective. Components of the core system project to an extended face system that contains separate regions for the interpretation and analysis of the different facial properties. The extended face regions have a wider role in processing stimuli other than faces. For example, the extended brain areas involved in facial expression recognition are also involved in processing other emotional stimuli from multiple sensory modalities. Research focusing on the extended face regions underlying facial expression recognition has identified a disproportionate, although not exclusive, role of certain brain regions in processing particular facial and vocal emotions, such as the amygdala's role in processing signals of fear [4–7] and insula's role in coding disgust [8–10]. By contrast, other brain areas, such as regions of prefrontal and somatosensory cortices seem to play a more general role in recognition of multiple facial expressions [11–13]; for reviews see [14,15]. The current paper explores how the function of components in the extended face system is affected by individual differences in personality traits that influence the manner in which we interact with facial signals of emotion and other emotional cues. In addition, we discuss that analyses that take account of individual differences in relevant personality dimensions reveal aspects of neural function that are not apparent when using the standard subtraction contrast method alone.

Neuroimaging techniques, such functional magnetic resonance imaging (fMRI), have been key in outlining the neural basis of facial expression processing. The vast majority of these studies have taken a group-based approach, in which the neural response to selected or multiple facial expressions is studied in a group of healthy individuals that are not pre-selected according to any specific criteria, other than generally being right-handed and having no history of neurological or psychiatric disorders. However, a wealth of cognitive and behavioural research has demonstrated that individual differences in people's personality can affect the manner in which they process emotional stimuli. For example, although anxiety is a normal emotional and physiological response to being threatened, individuals differ in the amount of anxiety they experience. The processing of threat-related stimuli has been investigated extensively in behavioural studies of individuals diagnosed with anxiety disorders and in non-clinical groups of participants with high and low levels of self-reported anxiety [16,17]. The results show that in both clinical and non-clinical populations, anxiety influences the behavioural response to stimuli conveying threat. In the case of facial expressions, high-anxious individuals show an increased propensity to orient their attention towards facial displays of fear and anger; an effect that is also found when the stimuli are presented outside of conscious awareness [18,19]. Similarly, additional research has shown that heightened levels of non-clinical anxiety can affect recognition of facial signals of fear [20]; for example, high-anxious individuals show increased sensitivity to low intensity exemplars of fearful faces in morphed facial continua; see also Surcinelli *et al*. [21].

Functional neuroimaging techniques, such as fMRI, are potentially equally as sensitive as the behavioural or cognitive paradigms used to study the effects of personality. Yet, the effect of individual variation in personality dimensions on brain function is frequently ignored or dismissed as noise in studies of healthy individuals. Recent research, however, shows that certain personality dimensions can account for a significant proportion of variance in the neural response to emotional stimuli; for example, the amygdala's response to facial signals of fear is significantly correlated with individual differences in anxiety [22–24]. This variation could also help explain why meta-analyses show that approximately 40 per cent of functional imaging studies addressing the neural response to facial expressions of fear fail to find a significant amygdalar response [25,26]. In other words, the presence or absence of an amygdalar response to facial signals of threat may depend on whether the participants' mean level of anxiety lies towards the upper or lower end of the anxiety range in the healthy population.

**Figure 1.**
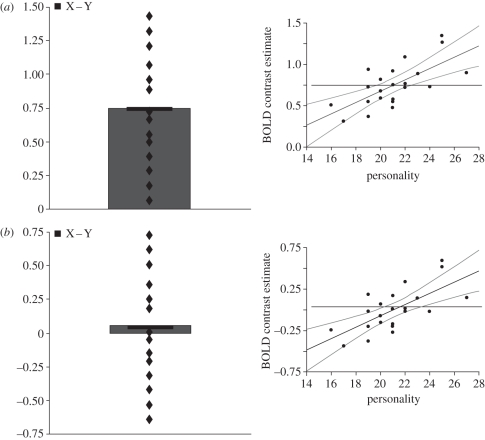
Two hypothetical datasets in which the neural response of a particular brain region to a stimulus is positively correlated with a personality variable. (*a*) A dataset for which a positive correlation is observed in conjunction with a group effect. (*b*) A dataset for which a positive correlation of equal magnitude is observed in the absence of a group effect.

As we go on to discuss, other personality dimensions are also important. However, this initial example serves to illustrate that the role of specific brain regions in processing facial expressions (or other stimuli) could be underestimated or missed without taking into account the influence of personality traits or other variables that have been shown to have a consistent impact on the processing of these stimuli. The graphic illustration in figure 1 underlines the utility of a correlational approach. In addition, it clarifies how it differs from the standard univariate approach in which the neural activation in response to one condition is subtracted from the activation associated with another. We apologise if the distinction is readily apparent to some readers, but having encountered three reviewers of previous experimental studies who asked how it was possible for a particular brain region to show a correlation with a personality variable in the absence of a group effect, we felt that others may also appreciate a short discussion of these two types of effects.

In brief, correlations and group (or main) effects are statistically distinct. For any given brain region, each can be observed in isolation or both can occur. Figure 1 illustrates two hypothetical datasets. In figure 1*a*, a correlation between a personality trait and the brain response to a particular contrast of interest (condition *X* − condition *Y*) occurs together with a significant main effect of this contrast. Figure 1*b* shows a second situation in which an equally robust correlation with a personality trait is found in the absence of a group main effect. The latter occurs because lower and higher scores on the personality dimension are associated with relative reductions and increases, respectively, in the neural response to the contrast of interest, producing an overall effect that does not statistically differ from zero. Similarly, it is possible for a brain region to show a significant overall reduction in the group analysis (owing to an increased response to *Y* relative to *X*); again, this can occur with or without a significant correlation with a personality trait or other variable. In summary, group effects and correlations provide different information on the response of a brain region to a particular stimulus. The former relates to the overall mean group effect, while the latter indicates whether a significant proportion of inter-subject variation in the neural response of that region can be accounted for by an independent variable. From figure 1*b*, it should be clear that a particular stimulus or experimental task might have a significant effect on the function of a given brain region, but that in some situations this could be missed by a standard subtraction contrast approach alone.

In the following sections, we discuss recent neuroimaging studies that demonstrate the influence of personality on the neural response to viewing emotional facial expressions. Reviews of the effects of personality traits on cognitive and behavioural indices of emotional processing can be found in other recent publications and are not reviewed here [16,27,28]. Our review is not intended to be exhaustive, but rather illustrative of the importance of considering individual variation in relevant personality dimensions. We acknowledge that individual differences in other factors, such as the participants' gender, handedness, age, race or genotype, have also been shown to affect the neural response to emotional stimuli. However, this work is beyond the scope of the current paper and interested readers should consult reviews by Cahill [29], Canli & Amin [30], Hamann & Canli [31], Wager *et al*. [32] and Hariri [33] for a discussion of these effects.

The paper is divided into three main sections. A first section describes the first work showing effects of personality on brain function before providing a more detailed account of how the neural response to certain facial expressions is related to individual differences in anxiety. A second section describes how individual differences in reward drive, a trait linked to aggression, affect the neural response to angry facial expressions. In a final section we consider limitations and criticisms of the correlation-based approach to functional neuroimaging and responses to these criticisms. We conclude that when used appropriately, neuroimaging analyses that factor in the relationship between relevant personality variables and neural activity provide a fruitful approach to understanding the neural basis of facial expression processing and social cognition in general.

## 2. Anxiety and the processing of facial signals of threat

As far as we are aware, the first study to show the influence of variation in personality on the neural response to emotional facial expressions in a non-clinical population was reported by Canli *et al*. [34]. The study showed that individual differences in participants' scores on a measure of extroversion were correlated with the left amygdala response to facial expressions of happiness relative to a neutral expression baseline. Since the left hemisphere has been implicated more in approach-related behaviour, the relationship with extroversion was interpreted as contributing to behaviour consistent with the social interactive style of extroverts. By contrast, the amygdala response to facial expressions of fear, anger and sadness showed no significant relationship with extroversion, nor with any of the other, so-called, ‘big-5’ major personality traits (neuroticism, openness, agreeableness and conscientiousness). However, the amygdala did show a significant overall increase in its response to fearful (versus neutral) expressions irrespective of personality (i.e. group effect), consistent with previous research [6,7,9].

Canli *et al*. [34] concluded that the group effect for fearful expressions (in the absence of any significant correlation) and amygdala correlation with extroversion for happy expressions (in the absence of a significant group effect) reflect two distinct processes in the amygdala. First, the consistent amygdala response to fearful faces is due to the universal importance of detecting cues to potentially dangerous events. Second, the variable response to happy expressions as function of extroversion reflects the more sociable nature of individuals scoring high on this scale. The first of these conclusions has not been consistently supported by subsequent research, however, which has demonstrated that the amygdala response to fearful faces is related to individual differences in participants' levels of anxiety [22–24,35,36].

Such a relationship with anxiety accords with neuroimaging investigations of clinical samples indicate that ‘hyper-responsivity’ of the amygdala may underlie many anxiety-related disorders including social anxiety disorder, post-traumatic stress disorder (PTSD) and specific phobia [37,38]. This has been linked to the central role of the amygdala in processing threat-related stimuli [39–41], including fearful faces [4–7]. However, functional neuroimaging studies of fear processing in the typical, non-clinical population have largely ignored the influence of anxiety, despite clear evidence of its influence on behavioural tasks in both clinical and non-clinical populations [16]. As already discussed, variation in the amygdala response to threatening stimuli as a function of individual differences in the non-clinical anxiety range could help explain why a significant proportion of neuroimaging studies fail to find a significant increase in amygdala activation to fearful faces [25,26]. Moreover, a relationship between amygdala activity and anxiety would demonstrate that a substantial degree of the variability in the amygdala response to facial signals of threat has a meaningful psychological basis. Consideration of relevant personality factors, such as anxiety, could therefore reveal insights into the function of the amygdala, and other components of the extended face system, that are not apparent when examining effects at a group-level that pool across a wide range of inter-subject variability. In other words, exploiting the amygdala–anxiety relationship offers the opportunity to gain a better understanding of the amygdala's role in processing different types of facial threat.

**Figure 2.**
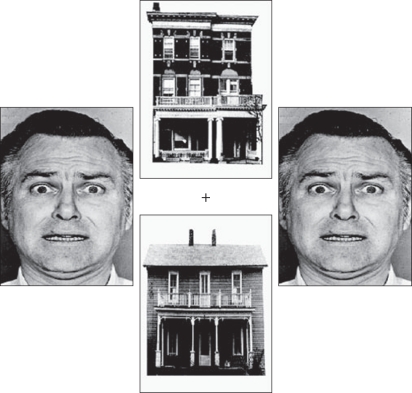
An example of a stimulus from the face/house paradigm. Two faces and two houses are presented in horizontal and vertical pairs around a central fixation cross. Participants are required to attend to either the horizontal or vertical images and to ignore the stimuli presented in the unattended location. Adapted from Bishop *et al*. [22].

**Figure 3.**
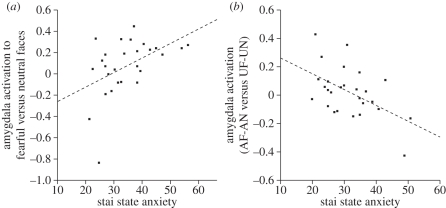
(*a*) State anxiety shows a positive relationship with the left amygdala response to fearful faces versus neutral faces across attended and unattended conditions. (*b*) Amygdala activity to attended fearful (AF) faces versus attended neutral (AN) faces relative to unattended fearful (UF) faces versus unattended neutral (UN) plotted as a function of state anxiety. Participants with higher state anxiety levels showed less attentional modulation of the amygdala response to fearful faces. Adapted from Bishop *et al*. [22].

To date, a number of studies have demonstrated that levels of anxiety in the non-clinical population are positively correlated with the amygdala response to fearful faces [22–24,35,36,42]. However, this relationship appears more evident when the fearful faces are unattended or presented outside of conscious awareness. For example, Bishop *et al*. [22] used a face/house paradigm, borrowed from previous work [43,44], in which participants were instructed to attend to pairs of faces or houses presented in the horizontal or vertical axes (figure 2). This enabled a comparison of the neural response to fearful and neutral faces when presented in an attended (‘attend faces’) or unattended (‘attend houses’) location. A group-level analysis revealed that the right amygdala showed an increased response to fearful relative to neutral faces across both attended and unattended conditions. Although this did not reach significance in the left amygdala, a further analysis showed that left amygdala response to this contrast was positively related to state anxiety (figure 3*a*). Moreover, although attention showed no significant interaction with emotional expression (fearful versus neutral) at a group level, the left amygdala response to this interaction was correlated with state anxiety. This reflected less attentional modulation of the amygdala response to fearful versus neutral faces in participants with higher anxiety levels (figure 3*b*). By contrast, both high- and low-anxious participants showed an increased amygdala response to fearful faces when attended.

A more recent study investigated the relationship between anxiety and attended/unattended fearful faces using a different paradigm [42]. Here, participants were shown a superimposed image of a house and face. When instructed to attend to the houses, high-anxious individuals showed an increased amygdala response to fearful faces. However, this effect was only found for female participants, suggesting a possible interaction with the participants' sex. The authors are careful to point out, however, that this sex difference may be owing to other moderating factors that might have differed between their male and female groups. For example, they note that depression can attenuate the attentional bias to threatening stimuli [45] and may, therefore, affect the relationship between the amygdala response and anxiety as well. In addition, as we go on to discuss, our own research has shown that the effects of anxiety on the amygdala response to threatening facial expressions survive factoring out any contribution of sex [36,46]. Hence, the idea that the relationship between anxiety and the amygdala response to threatening faces is specific to female participants seems unlikely, although it is important to consider this possibility in future research.

A third study by Etkin *et al*. [23] presented fearful faces inside and outside of the participants' conscious awareness by displaying backward masked faces at 33 ms and non-masked faces at 200 ms, respectively. Etkin *et al*. [23] found an increased amygdala response to consciously presented (200 ms) images of fearful faces that was independent of anxiety. However, amygdala activation to the unconscious presentations was only apparent when considering the influence of anxiety, with trait anxiety predicting an increased response in the basolateral amygdala.

Together, these studies suggest that the relationship between anxiety and the amygdala response is greater when fearful faces are unattended or presented outside of conscious awareness. This accords with behavioural evidence indicating that attentional capture in high-anxious participants is most effective when the stimuli are presented outside of awareness [18,19]. Increased amygdala activation to unattended fearful faces might suggest a role for the amygdala in evaluation or detection of pre-attentive threat. However, an alternative explanation is that the paradigms in these studies allowed limited residual perceptual resources to be allocated to unattended locations. The latter account is bolstered by the finding that high-anxious individuals show reduced recruitment of prefrontal control mechanisms in response to threat-related distractors relative to low-anxious participants [47]. Note also that Pessoa & Ungerleider [48] have demonstrated that a significant proportion of subjects can reliably detect masked presentations of fearful faces at a duration of 33 ms, which was used in the unconscious condition in Etkin *et al*.'s [23] study. Hence, this study may tap degraded perception, rather than unconscious perception in at least some subjects. To address this further, Bishop *et al*. [35] conducted a further experiment that used a letter search task of high or low perceptual load [49] superimposed on fearful or neutral face distractors. They found that high-anxious individuals showed an increased amygdala response to fearful distractors only for the low perceptual load condition. This result is difficult to reconcile with the proposal that the amygdala response to facial signals of fear is not gated by attention [43,50], or indeed that the amygdala response to facial signals of fear *in high-anxious participants* is not gated by attention.

**Figure 4.**
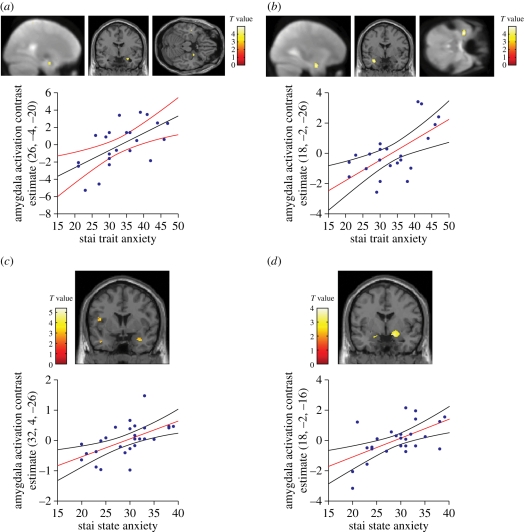
Trait anxiety shows a positive relationship with the amygdala response to (*a*) attended angry faces versus attended neutral faces, and (*b*) unattended fearful faces versus unattended neutral faces. State anxiety shows a positive relationship with the amygdala response to (*c*) direct gaze angry faces versus direct gaze neutral faces and (*d*) averted gaze fearful faces versus averted gaze neutral faces. Adapted from Ewbank *et al*. [36].

Like fearful facial expressions, angry faces also represent highly potent signals of threat, often rated as equally arousing and as unpleasant as fearful faces [51]. However, the influence of anxiety on the amygdala response to facial expressions other than fear has been somewhat neglected. This is a significant oversight given that facial signals of anger have been characterized as the prototypical stimulus for eliciting anxiety in low-ranking or socially submissive animals [52]. Using a similar face/house paradigm to that used by Bishop *et al*. [22], Ewbank *et al*. [24] investigated the effect of anxiety and attention on the neural response to both fearful and angry faces. Importantly, the exemplars of these two expressions were matched on ratings of arousal and valence; thus any effects could not be attributed to differences on these dimensions. While group-based contrasts revealed no difference in amygdala activation to anger and fear, regression analyses examining the influence of individual differences in anxiety revealed a differential effect of attention on the amygdala response to these two expressions. Relative to neutral faces, the amygdala response to unattended fearful faces was positively correlated with trait anxiety. Consistent with previous work, the relationship between anxiety and attended fearful faces was marginally less marked but achieved borderline significance with trait anxiety. By contrast, trait and state anxiety were positively related to the amygdala response to angry relative to neutral expressions, but only when attended (figure 4*a*,*b*); unattended angry faces showed no corresponding relationship with anxiety. Moreover, the different patterns for attended and unattended angry expressions were underlined by an additional analysis demonstrating that the right amygdala showed an increased response to attended versus unattended angry faces as a function of trait and state anxiety. These effects of anxiety persisted after factoring out any influence of the participants' sex and individual variation in social anxiety (i.e. fear of negative evaluation (FNE) [53]). Hence, these other factors do not seem to contribute to the effect.

One possible explanation for Ewbank *et al*.'s [24] findings is that angry and fearful faces represent two qualitatively different signals of threat [54,55]. While fearful faces signal the presence of threat or danger for which the source is undetermined, angry facial expressions signal a more direct and immediate threat and are used primarily in face-to-face encounters to control or manipulate others' behaviour [56,57]. Moreover, to fully evaluate the threat signalled by an angry face it is important to take the aggressor's identity, rank, sex, etc., into account, which requires focused attention. In contrast to anger, a fearful face may act as a potent cue to danger regardless of whether it is attended or not. However, fearful faces may be more effective when unattended—that is, prior to attentional capture and prior to knowledge of the possible source of the threat, or whether the signal may constitute a false alarm that should be ignored.

The aforementioned studies demonstrate that the observer's focus of attention has a clear influence on the neural response to facial signals of threat. However, additional research has shown that the *expresser's* direction of attention is an equally important factor that can affect the processing of facial signals of anger and fear. Behavioural studies report a consistent influence of gaze on the processing of angry faces, with angry faces being perceived as more threatening when their gaze is directed at, compared with away from, the observer [58–61]. Similarly, there is evidence that facial expression has a reciprocal influence on perception of gaze direction, with angry expressions causing gaze or gaze/head combinations to be more readily perceived as directed at the observer [46,62]. Across studies, the effect of gaze on the processing of fearful faces is less consistent than has been found for angry expressions. Some studies indicate that the processing of fearful faces is enhanced by averted relative to direct gaze [58,61], while other work reports enhanced processing for direct gaze fearful faces, or has found no significant influence of gaze on processing fearful faces [59,60,63]; see Bindemann *et al*. [59] for a detailed investigation of these effects.

Group-based fMRI studies investigating the influence of gaze on the neural response to facial signals of threat have reported mixed results [64–67]. An initial study by Adams *et al*. [65] found an increased amygdala response to averted gaze angry faces and direct gaze fearful faces, relative to the direct gaze angry and averted gaze fearful counterparts. By contrast, Sato *et al*. [67] reported an increased amygdala response to angry faces directed towards the observer, while Hadjikhani *et al*. [66] found an increased amygdala response to averted gaze fearful faces relative to direct gaze fearful faces. More recently, Straube *et al*. [64] showed a greater amygdala response to averted relative to direct gaze facial expressions that did not interact with the emotion displayed (anger, happy and neutral), and therefore appeared to reflect an effect of gaze direction, rather than an interaction between facial expression and gaze.

Given the important role of anxiety in evaluating facial signals of threat, it is reasonable to expect that anxiety might also play an important role in the interactive relationship between gaze and angry expressions. This is supported by recent behavioural evidence indicating that high-anxious participants show greater avoidance behaviour when faced with direct compared with averted gaze angry faces [68]. Similarly, Hess *et al*. [63] showed that angry faces gazing at, relative to away from, the observer produced increased self-reported anxiety in observers. By contrast, direct and averted fearful faces were rated as being equally anxiety-provoking. Thus, it is reasonable to predict that the relationship between the amygdala response and anxiety should be more evident for direct than averted gaze angry faces, whereas the relationship for fearful faces would be less dependent on gaze.

Consistent with this hypothesis, Ewbank *et al*. [36] found that state anxiety showed a positive linear relationship with the amygdala response to direct gaze angry faces relative to direct gaze neutral or averted gaze angry faces (figure 4*c*), but no corresponding effect for averted gaze angry faces. By contrast, for fearful faces, state anxiety predicted an increased amygdala response to both direct and averted gaze faces relative to their neutral face comparison conditions, although stronger evidence was found for the averted gaze condition (figure 4*d*). State anxiety was also correlated with the right amygdala/extended amygdala response to the interaction between emotion (anger and fear) and gaze (direct and averted), reflecting the fact that anxiety was associated with a gaze-dependent response to angry faces, but not fearful faces. The relationship between anxiety, expression and gaze direction was mirrored by behavioural ratings, which showed that increased anxiety was associated with increased ratings of anger for direct but not averted gaze angry faces, and with increased ratings of fear for both direct *and* averted gaze fearful faces.

These findings accord with other behavioural work showing that high-anxious participants find it harder to disengage from direct gaze angry faces and show enhanced attentional-orienting from averted gaze fearful faces [69,70]. In addition, they fit with the observation that direct gaze angry faces are rated as more angry, whereas the effect of gaze on the perception of fearful faces is less consistent [59,60,63]. Ewbank *et al*.'s [36] findings also correspond extremely well with the results of our earlier neuroimaging study examining the interactive effects of facial expression (anger and fear) and the participants' focus of attention and levels of anxiety. Recall that this showed that anxiety was positively related to the amygdala response to attended, but not unattended, angry faces. By contrast, anxiety showed a positive relationship with the amygdala response to both attended and unattended fearful faces, with a marginally larger effect for unattended fear; see also [22]. Thus, the amygdala response to angry faces shows a positive monotonic relationship with anxiety when the faces are attended by, or their gaze is directed towards, the participants. By contrast, the positive relationship between anxiety and the amygdala response to fearful faces may be less dependent on gaze direction, but there is a bias towards more marked effects for unattended faces in the studies to date.

The observation that the relationship between anxiety and the amygdala response to threat is differentially influenced by the attention and gaze for facial signals of anger and fear accords with the different nature of threat signalled by these expressions. Angry expressions afford a maximal state of vigilance when the threat is directed at and attended by the observer, owing to their maximal potential danger in this form. By contrast, the threat signalled by facial expressions of fear appears less dependent on gaze direction. In addition, as already discussed, the greater relationship between anxiety and unattended facial expressions may reflect the fact that these signals are perceived as more aversive prior to establishing the source of the threat, or prior to determining whether the signal constitutes a potential false alarm.

The results of these experiments also suggest that the amygdala response is not selective for a particular expression, nor related to the arousal value of the face, but instead reflects the degree of threat, or more specifically, the relevance of the signal to the observer. These findings, therefore, support the proposal that the amygdala constitutes an evolved system for appraisal of self-relevance, primarily involved in responding to stimuli or events that are of high personal relevance [71,72]; see also Adolphs [73] and Ewbank *et al*. [74]. As such, the relationship between anxiety and the amygdala response to threat-related facial expressions is difficult to reconcile with the proposal that the amygdala is involved in coding signals of ambiguity and responds more to ambiguous threat [55]. In other words, increasing anxiety is not associated with an increased amygdala response to signals of ambiguous threat (e.g. averted gaze angry faces), but instead shows an increased response to unambiguous threat (e.g. direct gaze angry faces).

**Figure 5.**
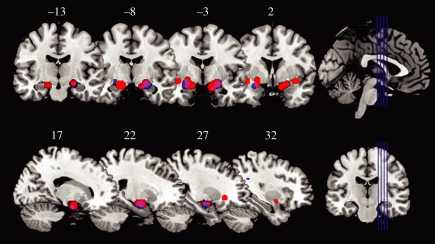
The activation likelihood estimation (ALE) method was used to identify brain areas showing correlations between anxiety and change in BOLD signal while viewing threat-related facial expressions (fear and anger) across 162 participants from six studies. The most consistently activated areas are displayed on coronal (top) and sagittal (bottom) sections of a T1 MNI template. Correlations with state anxiety are shown in red, correlations with trait anxiety in blue and areas of overlap appear in purple. ALE was performed using GingerALE software [75]. The ALE method quantifies the degree of correspondence in three-dimensional-stereotactic coordinates of activation foci across functional neuroimaging studies, and uses significance thresholds to create statistically defensible conclusions (i.e. inter-study consistencies) about the summarized data. Significantly activated regions (*p* < 0.05 false discovery rate (FDR) corrected) show a positive correlation with anxiety when viewing angry or fearful faces. The following studies were included in the ALE analysis [22–24,35,36,42].

Overall, the work reviewed above demonstrates that individual variation in anxiety within a non-clinical population shows a consistent positive relationship with the neural response to facial signals of threat. To determine the reliability of these anxiety-related brain activations, we have summarized the results of the aforementioned studies using activation likelihood estimations (ALEs) [75]. Here, we included all fMRI studies reporting a correlation between state or trait anxiety and the neural response to fearful or angry faces in non-clinical populations. According to the ALE procedure, the most statistically reliable activations were identified in bilateral amygdalae (figure 5), further supporting the relationship between anxiety and this region. A full list of regions showing significant relationships with both state or trait anxiety can be seen in table 1.

**Table 1.** Centres of clusters showing a correlation with state or trait anxiety when viewing angry or fearful faces across 162 participants in six studies; significant at *p* < 0.05 false discovery rate (FDR) corrected. Includes all reported contrasts comparing activation to fearful or angry facial expressions with that observed for a neutral face or another facial expression.

MNI coordinates				
*x*	*y*	*z*	volume (mm^3^)	region
24	−6	−14	4752	right amygdala
−26	−2	−14	2824	left amygdala
48	−44	6	448	superior temporal sulcus/mid temporal gyrus
34	−46	28	288	inferior parietal lobe
28	24	34	128	middle frontal gyrus
−48	−42	−8	120	fusiform gyrus
−16	−40	44	120	posterior cingulate gyrus
−38	0	−10	104	left mid insula

It is notable that correlations with state anxiety are located in slightly more dorsal and medial regions of the amygdala in comparison to correlations with trait anxiety, which are associated with slightly more lateral regions. The central nucleus of the amygdala is located in the dorsal section of the primate brain and projects to cortical and brainstem regions involved in autonomic arousal [76]. By contrast, the basolateral amygdala (lateral section of the amygdala) projects to the central nucleus, and is thought to be critical for the acquisition of learned-fear responses [77]. It is also of note that the state-related activation extends beyond the dorsal amygdala into the extended amygdala [78,79]. This includes the substantia innominata, which is sensitive to ‘arousing’ stimuli [80,81]. Thus, one possible interpretation is that responses correlating with state anxiety reflect increases in arousal, while responses correlating with trait anxiety correspond to enhanced processing (or encoding) of facial signals of threat. However, considering the relatively small number of studies that have examined the relationship between anxiety and the amygdala response, and the strong correlation between state and trait anxiety, further studies will be necessary to establish whether there are any reliable differences between the amygdala responses to these two measures of anxiety. Any neuroanatomical distinction is further tempered by the fact that all but one of the studies summarized in figure 5 used a relatively standard acquisition sequence. High-resolution imaging, however, will be necessary to draw conclusions concerning the relative contribution of different sections of the amygdaloid complex to state and trait anxiety. Note also, that since similar numbers of studies have found correlations between state and trait anxiety and the amygdala response to facial signals of threat, we find little support for the suggestion that amygdala activity to these stimuli is primarily related to state anxiety [82].

In summary, a number of studies have reported a correlation between neural activity and individual variation in self-reported anxiety (both state and trait) when viewing facial signals of threat, particularly in the amygdala. A common theme across these studies is that a particular effect or interaction is not apparent, or less apparent, when using a standard univariate approach in which mean activity to two or more conditions is compared in the group as a whole. Rather, differential effects of attention, facial expression and eye gaze were more evident when factoring in the contribution of individual differences in anxiety. Thus, taking account of individual variation in anxiety has helped untangle the neural mechanisms underlying the processing of different forms of facial threat in the healthy population, both in terms of understanding the influence of the observer's focus of attention and the faces' gaze direction. Next, we consider research showing that the neural response to angry expressions is also affected by participants' sensitivity to reward.

## 3. The behavioural activation system and the processing of facial signals of aggression

Facial expressions of anger are manifest signals of threat for the individual at whom they are directed. However, the manner in which people react to such stimuli is significantly influenced by differences in personality traits and therefore differs from one individual to the next. For example, as discussed in §2, high-anxious participants may be more intimidated when facing social threats and consequently withdraw. In contrast, certain other individuals may feel provoked by displays of aggression and respond in a hostile manner. Behavioural research has indicated that these latter individuals often score high on a personality measure that assesses sensitivity to reward, such as the behavioural approach system (BAS) scale [83].^1^

Although the relationship between reward and aggression is not immediately apparent, comparative research has demonstrated the adaptive function of aggression in obtaining or maintaining valued resources such as food, reproductive partners, territory and social status [57]. Furthermore, omission or termination of a reward is a powerful instigator of aggressive behaviour in both human and non-human species [84–87]. Similarly, it is of note that the aggression seen in psychopathy—a condition associated with high BAS activity [88,89]—is frequently goal directed and linked to the gain of monetary and sexual rewards or social status [90].

A number of studies have demonstrated a relationship between BAS and aggression. Harmon-Jones [91] reported positive associations between BAS and trait anger, and between BAS and a self-report measure of physical aggression. Similarly, Smits & Kuppens [92] found an association between BAS and trait anger, and between BAS and reports of both physical and verbal aggressions. In a frustrative non-reward study, Carver [84] induced anger by using a task manipulation that caused participants to lose a reward and found a positive relationship between BAS and experienced frustration. Wingrove & Bond [85] also used an active behavioural task in which participants played a computer game that required cooperation with a partner. Unbeknown to participants, most trials were rigged to cause them to fail, resulting in anger and frustration. Measures of situational quarrelsomeness, resentfulness, discontent and hostility in this study were correlated with two scales of the BAS measure. For a more detailed review of these studies, see Carver & Harmon-Jones [27].

The relationship between BAS and anger also extends to processing facial expressions of anger. Putman *et al*. [93] showed a positive correlation between BAS and participants' response times to identify the colour of a transparent wash placed over masked presentations of facial signals of anger in a colour Stroop task. This was attributed to high BAS participants' increased attention to angry faces, perhaps resulting from their tendency to interpret these facial displays as signals of provocation or social challenge [94,95]. A similar effect has been observed in high trait anger participants [95].

**Figure 6.**
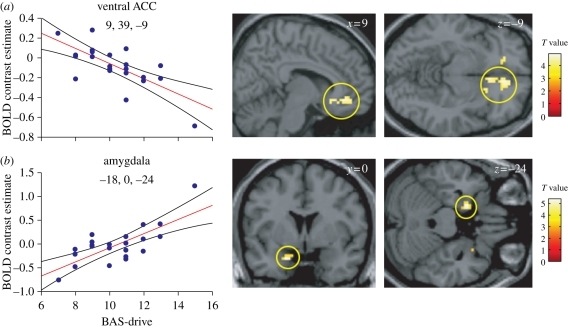
(*a*) A negative correlation between the ventral anterior cingulate response to facial signals of aggression (relative to neutral expressions) and BAS-drive. (*b*) A positive correlation between the amygdala response to facial signals of aggression (relative to neutral expressions) and BAS-drive. The scatter plots show BOLD signal change for peak-activated voxels for each contrast plotted as a function of participants' BAS-drive scores. Regression lines and 95% confidence intervals are shown. Adapted from Beaver *et al*. [96].

In view of these behavioural findings, we have examined how neurophysiological responses to viewing facial signals of aggression are related to inter-subject variability in BAS. In a first fMRI experiment, we found that the BAS-drive component of this personality trait, measuring participants' drive or motivation to gain a reward or goal, was correlated with increased amygdala activation and a decreased ventromedial prefrontal cortex (vmPFC, ventral anterior cingulate cortex (vACC)) response when participants viewed pictures of angry (relative to neutral or sad) faces (figure 6) [96]. Interestingly, the same inverse relationship between the amygdala and vmPFC has been observed when evoking subjective experience of anger [97]. These findings also accord with animal research that emphasizes the role of the amygdala in triggering the negative effect associated with anger and the role of vmPFC in control or regulation of emotion, with anger associated with increased negative effect and decreased emotional control.

The vmPFC and amygdala are tightly interconnected regions [98,99], so we reasoned that a measure of the dynamic interplay between them would represent a better index of the brain mechanisms underlying the processing of facial signals of aggression and their relationship with BAS-drive. Thus, in a second fMRI study, we specifically addressed the change in *effective connectivity* or coupling between the vmPFC and amygdala while participants viewed angry relative to neutral faces [100]. Effective connectivity was measured using two techniques—psycho-physiological interactions (PPIs) and dynamic causal modelling (DCM) [101,102]. PPI investigates how a change in a psychological context (e.g. viewing angry versus neutral faces) affects the connectivity (i.e. the correlation) between a ‘source’ region (here the amygdala) and the rest of the brain. DCM is an alternative approach to effective connectivity analysis that enables inferences about the directionality of causal connections between regions (e.g. a change in connectivity from region A to B or vice versa) within a hypothesis-driven anatomical model.

**Figure 7.**
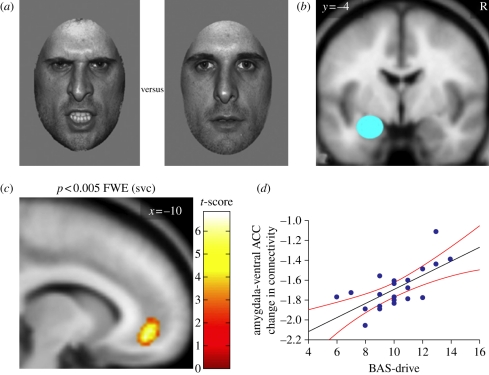
(*a*) Examples of the angry and neutral faces. (*b*) Amygdala source region for the psycho-physiological interaction (PPI). R, right hemisphere. (*c*) PPI statistical parametrical map (SPM) showing that, when viewing angry versus neutral faces, the ventral ACC shows a change in connectivity with the amygdala (source region) that is correlated with individual differences in BAS-drive. (*d*) Data plot for the PPI shown in panel (*c*). Participants with higher BAS-drive scores show decreased negative connectivity between the ventral ACC and the amygdala. The regression line and the 95% confidence intervals are shown. Adapted from Passamonti *et al*. [100].

The results of the PPI analysis were remarkably specific. Across the group as a whole (i.e. irrespective of individual differences in BAS-drive), the amygdala showed a borderline negative connectivity with the left vmPFC (vACC) [100]. However, our hypothesis was that the magnitude of this effect might reflect systematic individual differences in BAS-drive personality. Consistent with this, BAS-drive was highly correlated with the change in connectivity between the amygdala and vmPFC (vACC), with high-BAS participants presenting reduced negative connectivity in response to viewing angry relative to neutral faces relative to low-BAS participants (figure 7). Using DCM, we explored the directionality of the effect, and found significant changes in connectivity between the amygdala and vACC, and vice versa, as a function of viewing angry versus neutral faces across the group as a whole. However, the effect of BAS-drive on this connectivity was restricted to projections from the vACC to the amygdala; connectivity in the opposite direction was unrelated to BAS-drive.

With respect to the research discussed previously showing that anxiety can also affect the amygdalar response to facial signals of aggression, a separate connectivity analysis of the same data examined the influence of individual differences in state anxiety with PPI [100]. This identified a change in connectivity between the amygdala and dorsal anterior cingulate, and between the amygdala and ventrolateral prefrontal cortex (vlPFC). In addition to the amygdala, both frontal regions have also been associated with individual differences in anxiety in selected previous studies [35,47]. Although we did not investigate this network further using DCM, it is of interest that neither state nor trait anxiety (or the behavioural inhibition scale (BIS), which is also related to anxiety) was correlated with the change in connectivity between the vACC and amygdala identified by the DCM analysis investigating the relationship with BAS-drive. Hence, investigating the influence of individual differences in BAS-drive and anxiety in the context of a connectivity analysis allowed us to identify two distinct networks: one potentially underlying the increased antagonistic response to angry expressions associated with anger-prone individuals, and the other, the increased withdrawal response to these signals associated with anxiety-prone individuals.

An additional interesting observation from the DCM analysis was that the optimal neuroanatomical model for processing angry and neutral expressions included simultaneous inputs of facial information into both the amygdala and vACC (‘parallel model’), rather than an input to either of the two regions alone (‘serial models’). This ‘parallel’ model of processing facial expressions accords with electrophysiological recordings in non-human primates [103,104] and humans [105] showing that the amygdala and vmPFC/vACC respond quickly and within approximately the same time-scale window (approx. 120–220 ms) to faces. Similarly, electroencephalographic (EEG) recordings show that frontal and frontocentral regions show a more positive (or less negative) response to emotional relative to neutral faces between 120 and 300 ms [106–108].

fMRI is known to have poor temporal resolution and so inferences about the timing of effects are limited from our fMRI experiments examining the influence of BAS. To address the temporal properties of the effect of BAS-drive on the frontal response to facial expressions, we conducted a further experiment using EEG recordings in separate groups of high-BAS-drive and low-BAS-drive participants [109]. On the basis of previous findings showing a reduced vmPFC response to angry compared with sad or neutral expressions in participants with increased BAS-drive [96], we hypothesized that high-BAS-drive individuals would also show a selective reduction in the frontal event-related potentials (ERPs) to angry faces relative to sad and neutral faces. Following earlier research [106,107], we focused on the frontal response within two time windows (130–200 ms and 200–300 ms post-stimulus onset). Consistent with this previous research, the first time window (130–200 ms) showed enhanced positive ERPs for emotional (angry or sad) relative to neutral faces, but these ERPs showed no significant relationship with BAS-drive. A similar group effect of emotion was also observed for the second time window (200–300 ms); however, this was qualified by an interaction with BAS-drive. Further analyses showed that the low-BAS-drive group showed significant differences between angry and neutral or sad faces, whereas no such differences were found for the high-BAS-drive group. Thus, the effect of personality on the frontocentral response to facial expressions was restricted to the second time window.

The findings of the EEG study accord with the conclusions from our connectivity analysis that there are at least two successive stages in the processing of facial expressions in the vmPFC and the amygdala [100]. A first phase (up to about 200 ms) may reflect encoding of stimulus significance, resulting in a rapid categorization of the stimulus as emotional or not. This stage may rely on interactions between vmPFC and amygdala that are unaffected by differences in personality. During a second phase (starting around 200 ms), interactive effects of facial emotion and personality (BAS-drive) take place. Here, differences in the subjective relevance or salience of affective stimuli are computed according to ‘person-specific’ criteria that assist in adaptive ongoing behaviour. During this second stage, the reduction of the vmPFC response to angry faces in high-BAS-drive individuals may result in a diminished ability of the vmPFC to downregulate amygdala activity in response to angry faces in high-BAS-drive individuals. The former is evidenced by a decreased local fMRI response and less negative (more positive) fronto-central ERP activity, while the decreased downregulation is evidenced by reduced negative connectivity from the vmPFC to the amygdala with increasing BAS-drive, but not vice versa.

In summary, our studies addressing the influence of BAS on processing angry faces identify a specific network and time window for the interaction between a personality factor linked with aggression and the processing of these facial signals. In addition, they provide a potential neurobiological account for the influence of BAS-drive on aggression in general. The effects of BAS-drive do not simply reflect variation in general emotional arousal, because levels of anxiety and threat-sensitivity were correlated with activity in other regions, including the dorsal ACC—an area implicated in fear conditioning [110] and anticipation of aversive events [111]—which showed altered activation or connectivity with the amygdala as a function of anxiety [96,100]. Consequently, our findings may also provide a potential neural account of why angry faces are interpreted as provocative (producing an aggressive response) or frightening (producing a withdrawal response) according to an individual's personality/temperament and brain areas engaged.

## 4. Individual differences and functional imaging

This review has outlined a number of studies reporting a consistent relationship between behavioural measures of personality and the neural processing of particular facial expressions. However, this type of research, and investigations of the relationship between individual differences and neural activity in general, has recently prompted a lively debate. One group of researchers have questioned the statistical validity of many findings using this approach, and suggest that the relationship between behavioural measures and brain activation has been over-inflated [112]. Vul *et al*. [112] argue that when the reliability of both fMRI and the behavioural measures used to assess individual differences are considered, the reported correlations between the two appear ‘puzzlingly high’. They propose that this over-inflation is the result of ‘non-independence’ errors, and that such errors are widespread in fMRI studies of individual differences.

In a non-independent analysis of neuroimaging data, voxels are selected on the basis of achieving a particular threshold in an initial analysis and then a secondary analysis is performed on the data from those same voxels. Thus, the selection procedure is not independent of the relevant measure. In the case of regression analyses investigating individual differences in neuroimaging, Vul *et al*. [112] argue that a relationship between a behavioural measure and brain activity is often the selection criterion, while the correlation coefficient(s) between the behavioural measure and the selected voxel(s) constitute(s) the secondary statistic. Insufficient correction for multiple comparisons means that a greater number of voxels showing a correlation with noise will pass the statistical threshold (false positives), and the results of the secondary analysis are guaranteed to be significant. Vul *et al*. discuss that in this way, non-independent analyses result in inflation of the correlation coefficients.

Vul *et al*. [112] focused their paper on functional neuroimaging studies in social cognitive neuroscience. As others have pointed out, this type of error is not unique to social neuroscience, and has been highlighted in previous papers addressing the analysis of neuroimaging data and other multivariate datasets [113–115]. However, Vul *et al*. [112] argued that a number of high-profile publications in social neuroscience investigating individual differences are guilty of non-independence errors and therefore concentrated on these studies. Some associated commentaries and responses [113,116,117] have contested Vul *et al*.'s criticisms, arguing that the problem is much less widespread or severe than Vul *et al*. imply. Others emphasize that the problem is not unique to fMRI, or discuss lessons learned from correlational analyses of other multivariate datasets or other reasons why correlation coefficients can be inflated [114,118]. It is impossible to do justice to the numerous arguments outlined in these papers in the following sections, and we recommend that interested readers should consult these papers themselves.

To illustrate the non-independence error, Vul *et al*. [112] present the example of temperature readings taken from a weather station that happens to predict daily changes in the value of a set of stocks on the New York stock exchange (NYSE) with an average correlation coefficient (*r*) of −0.87. The selected stocks were those whose correlation coefficient exceeded a particular threshold after computing the correlation between the temperature readings of the weather station and each of the 3315 stocks on the NYSE. Some of the stocks were sure to be correlated with temperature measurements simply by chance. So the selected stocks were guaranteed to show a relatively high average correlation. However, Nichols & Poline [113] point out that such an example is based entirely on a null hypothesis argument (i.e. all of the relationships reflect noise). By contrast, they argue that correcting for multiple comparisons (e.g. *p* < 0.05 family-wise error (FWE) correction threshold)—a recommended procedure for analysis of neuroimaging data [119]—should result in no false positives at all with 95% confidence. Thus, significantly active voxels at *corrected* thresholds are likely to reflect true positives.

Even when controlling for multiple comparisons, a valid concern of Vul *et al*. [112] still remains, namely that a number of readers are likely to consider the correlation magnitude as important. Nichols & Poline [113] suggest that correlations should be reported for what they are (i.e. suprathreshold values that are post hoc measures of significance, uncorrected for multiple testing). In analyses of neuroimaging datasets, they are equivalent to raw *t*-statistics, which are often reported together with corrected *p*-values. In short, they should not be interpreted in the same way as correlation coefficients examining the relationship between just two variables; neither should they be interpreted as an estimate of the effect size. However, the principal concern of Vul *et al*. [112] is important, in that the magnitude of the correlation between neural activity and a given behavioural measure should not be inferred as representing an index of the true relationship between the two. In this respect, please note that none of the studies that we have reviewed in this paper has relied on the uncorrected *p*-value of a correlation coefficient as the measure of statistical significance.^2^ Rather, all but one of these studies, reported maximal raw *t*- or *z*-statistics of voxel clusters together with *p*-values corrected for multiple comparisons using small volume (e.g. FWE) correction. Moreover, figure 5 and table 1 illustrate that the amygdala response to facial signals of threat showed a highly consistent relationship with anxiety, which was present in all studies reported. Similarly, the neural response to facial signals of aggression in the amygdala and vmPFC showed a significant relationship with BAS-drive using a number of different neuroimaging techniques. Nonetheless, the correlation coefficients in these studies may reflect an inflated estimate of the true magnitude of the relationship.

According to Vul *et al*. [112], the strength of the relationship between the brain activation and a behavioural measure is further misrepresented through the inclusion of ‘visually pleasing’ scatter plots, which show the relationship between a behavioural measure and the peak voxel of a significant cluster. However, like others, we would argue that the inclusion of a scatter plot is an essential aspect of studies investigating individual differences, not least because an apparent ‘linear relationship’ between two variables can result from clearly different data distributions [116,117]. Scatter plots are needed not only to demonstrate that the correlation is not driven by one or two outliers, but also can reveal the presence of a bimodal or even a quadratic distribution, for example [120].^3^

Vul *et al*. [112] suggest two solutions to the problems they highlight. The first is to use an independently defined region of interest (ROI), such as an anatomically defined ROI or a functionally defined ROI. For example, a comparison of fearful and neutral faces in a separate ‘functional localizer scan’ might be used to identify selected voxels in the amygdala that are responsive to fearful expressions. The signal in these voxels for the contrast of interest in the main experiment can be extracted and correlated with a specific behavioural variable. As illustrated in figure 1, however, correlations between a behavioural measure and the brain activity of a particular region can be observed in the absence of a main group effect in the same region. Consistent with this, we also discussed that 40 per cent of experiments examining brain activation to fearful facial expressions have failed to find a significant amygdala response [25,26]. In contrast, all of the studies we have reported found a significant relationship between anxiety and amygdala activation to fearful faces, although we accept that more studies addressing the latter are required.

As a further alternative, Vul *et al*. [112] propose that researchers split their datasets into two halves. One half can be used to identify the voxels that show a correlation between a behavioural measure and brain activation to a particular contrast. The remaining data are then used to test whether the selected voxels show a similar relationship in a separate analysis. However, Poldrack & Mumford [117] caution that any between-subject variance will produce a correlation between runs, making this approach non-independent. Moreover, they point out that using fewer data will result in less statistical power; but see Vul *et al*. [121] for a response.

One of the commentaries by Yarkoni [118] makes an important contribution to the debate by arguing that although correlation coefficients in fMRI studies may indeed be inflated, the principal cause is different to that suggested by Vul *et al*. In essence, Yarkoni highlights that the relatively small sample sizes typical of fMRI research require high correlation coefficients to achieve the stringent alpha-correction levels used in fMRI. Hence, significant *r*-values will always be large in restricted sample sizes. In addition, they point out that as sample sizes increase, effect sizes (including correlation coefficients) typically decrease. However, larger samples provide a truer estimate of the actual population effect size. Another adverse consequence of limited power is type II error, that is, the failure to detect real effects. Of course this is a problem for all research, but the problem is exacerbated for correlational analyses because they typically require large sample sizes to achieve adequate power.

Yarkoni [118] is careful to point out, however, that the limitations he identifies are based on the assumption that the true size of correlation coefficients observed in fMRI is approximately the same as those found in behavioural research. In the absence of any evidence to the contrary, this seems a reasonable starting point. But it is interesting to note that the correlations between the amygdala and anxiety we have reported are consistent across studies. If the magnitude of the population coefficient is relatively low, however, then we might expect that a significant relationship between anxiety and the amygdala response would be found in fewer studies than observed given their relatively restricted sample sizes in comparison with behavioural research. Note also, that given the concerns relating to type II error, the results of these studies cannot be taken as evidence that the relationship between anxiety and the brain is somehow ‘selective’ to the amygdala and other brain areas summarized in table 1. Rather, they indicate the important and reliable influence that anxiety has on the neural response of these areas to facial signals of threat. Better estimates of the population effect will require larger sample sizes. Indeed, as Yarkoni points out, larger samples are the obvious solution to the problems he identifies.

As with all fields of science, the reliability of the relationship between variables can only be established through independent testing and replication. This review has sought to highlight the reliability of the relationship between behavioural measures of specific personality traits and the neural response to certain facial expressions. In particular, we show that the relationship between anxiety and the amygdala response to facial signals of threat is found across a number of studies (see ALE analysis reported above; figure 5). Similarly, initial research shows that a relationship between BAS-drive and the neural response to facial signals of aggression can be detected using different neuroimaging techniques (EEG recordings and regression analyses and effective connectivity analyses of fMRI data).

Vul *et al*. [112] and others have highlighted some weaknesses in the study of individual differences, and future studies should consider the concerns they have raised. In addition to the alternative methods of analysis suggested by Vul *et al*., Yarkoni [118] suggests that where *r*-values are presented they should be accompanied by confidence intervals to indicate their reliability. Failing this, the correlation coefficient should probably be omitted and the *z*- or *t*-statistic and corrected *p*-value reported alone. In this way, any confusion regarding whether a secondary non-independent analysis has been conducted can be avoided and the magnitude of the relationship not over-emphasized. Indeed, correlation coefficients are often calculated by the experimenters in post hoc analyses, rather than being provided by the analysis software by default. However, it is important to stress once again that none of the studies we have considered has drawn conclusions on the basis of secondary statistics. Moreover, following the first demonstration of a relationship between anxiety and the amygdala response to facial signals of threat, all subsequent studies confirmed their *a priori* hypothesis that the amygdala would show a similar effect.

In conclusion, identifying a relationship between certain personality dimensions and the neural response to facial signals of emotion is informative at two levels. First, it provides increased understanding of the neural basis of the personality variables. Second, the cognitive neuroscientist can exploit these relationships to improve our knowledge of the neural basis of processing facial expressions and emotional stimuli in general. Variables such as anxiety vary continuously across both healthy and clinical populations. So research that takes account of this variation, by including a correlational analysis, will be better placed to delineate the neural mechanisms of emotional processing than studies that rely on the standard subtraction contrast procedure alone. The approach is not restricted to investigations of emotional dimensions, however. There is good evidence that a measure of severity of autism symptoms, the autism-spectrum quotient (AQ) [122] varies continuously across both typical and clinical populations, and that it predicts performance in certain behavioural tasks that are impaired in people with autism [123–125]. In addition, initial work in typical, healthy participants shows that AQ scores correlate with the structure and neural response of the posterior superior temporal sulcus, a brain region implicated in social processing and autism [126]. Numerous studies have also investigated how task performance relates to neural activation. This includes recent work showing that performance on face perception tasks predicts the structure and neural responses of temporal lobe regions in congenital prosopagnosics and controls [127–129]. It is hoped that future work examining the influence of individual differences may provide additional insights into other aspects of face perception. In addition, these studies may help reveal that apparent inconsistencies in the literature are attributable to variation in relevant psychological dimensions, rather than meaningless noise.

## Endnotes

1 The BAS forms one component of the behavioural inhibition scale (BIS)/behavioural activation scale (BAS); a questionnaire that assesses personality measures related to reward sensitivity (BAS) and to behavioural inhibition/anxiety (BIS) [83]. The BAS includes three subscales assessing strong pursuit of appetitive goals (BAS-drive) (e.g. ‘I go out of my way to get things I want’), the inclination to seek out new rewarding situations (BAS fun-seeking) (e.g. ‘I'm always willing to try something new if I think it will be fun’), and positive effect/excitability (BAS reward responsiveness) (e.g. ‘When good things happen to me, it affects me strongly’).

2 Some of the papers discussed reported correlation coefficients, but these were presented in addition to analyses in which the statistical significance of the relationship was first verified using a correction for multiple comparisons.

3 As a scatter plot showing a continuous linear relationship with neural activity does not provide evidence for a robust linear relationship in itself [117], however, clear evidence of the alternatives (outliers, bimodal distribution, etc.) would raise cause for concern.
